# Structural features in common of HBV and HIV-1 resistance against chirally-distinct nucleoside analogues entecavir and lamivudine

**DOI:** 10.1038/s41598-020-59775-w

**Published:** 2020-02-20

**Authors:** Yoshiaki Yasutake, Shin-ichiro Hattori, Noriko Tamura, Kouki Matsuda, Satoru Kohgo, Kenji Maeda, Hiroaki Mitsuya

**Affiliations:** 1 Bioproduction Research Institute, National Institute of Advanced Industrial Science and Technology (AIST), Sapporo, 062-8517 Japan; 2 Computational Bio Big-Data Open Innovation Laboratory (CBBD-OIL), AIST, Tokyo, 169-8555 Japan; 3 National Center for Global Health and Medicine Research Institute, Tokyo, 162-8655 Japan; 4 Faculty of Pharmaceutical Sciences, Sojo University, Kumamoto, 860-0082 Japan; 5 Experimental Retrovirology Section, HIV and AIDS Malignancy Branch, National Cancer Institute, National Institutes of Health, Bethesda, MD 20892 USA; 6 Department of Clinical Sciences, Kumamoto University Hospital, Kumamoto, 860-8556 Japan

**Keywords:** Antimicrobial resistance, Antivirals, Hepatitis B virus, X-ray crystallography

## Abstract

Chronic hepatitis B virus (HBV) infection is a major public health problem that affects millions of people worldwide. Nucleoside analogue reverse transcriptase (RT) inhibitors, such as entecavir (ETV) and lamivudine (3TC), serve as crucial anti-HBV drugs. However, structural studies of HBV RT have been hampered due to its unexpectedly poor solubility. Here, we show that human immunodeficiency virus type-1 (HIV-1) with HBV-associated amino acid substitutions Y115F/F116Y/Q151M in its RT (HIV^Y115F/F116Y/Q151M^) is highly susceptible to ETV and 3TC. Additionally, we experimentally simulated previously reported ETV/3TC resistance for HBV using HIV^Y115F/F116Y/Q151M^ with F160M/M184V (L180M/M204V in HBV RT) substituted. We determined crystal structures for HIV-1 RT^Y115F/F116Y/Q151M^:DNA complexed with 3TC-triphosphate (3TC-TP)/ETV-triphosphate (ETV-TP)/dCTP/dGTP. These structures revealed an atypically tight binding conformation of 3TC-TP, where the Met184 side-chain is pushed away by the oxathiolane of 3TC-TP and exocyclic methylene of ETV-TP. Structural analysis of RT^Y115F/F116Y/Q151M/F160M/M184V^:DNA:3TC-TP also demonstrated that the loosely bound 3TC-TP is misaligned at the active site to prevent a steric clash with the side chain γ-methyl of Val184. These findings shed light on the common structural mechanism of HBV and HIV-1 resistance to 3TC and ETV and should aid in the design of new agents to overcome drug resistance to 3TC and ETV.

## Introduction

Hepatitis B virus (HBV) is a major pathogen causing human liver diseases such as chronic hepatitis B, liver cirrhosis, and hepatocellular carcinoma^1^, and affects approximately 250 million worldwide, resulting in nearly 1 million deaths per year^2^. Although HBV is a DNA virus comprised of a compact 3.2 kb genome, the polymerase (Pol) gene that spans three-quarters of the HBV genome contains reverse transcriptase (RT) and is essential for viral replication, as HBV replicates via a pre-genomic RNA intermediate^3,4^. HBV Pol is a multifunctional protein with molecular weight of ~90 kDa, and is composed of four distinct domains: terminal protein (TP), spacer, RT, and ribonuclease H (RH). Duck HBV Pol has provided a model to study a unique reverse transcription initiation mechanism whereby HBV Pol binds to a signal sequence termed ε on the template pre-genomic RNA. Reverse transcription is then initiated through a conserved Tyr residue in the TP domain acting as the sole protein primer^5,6^. The resultant covalently-linked ribonucleoprotein is simultaneously packaged with HBV core protein to form nucleocapsid core particles. Recombinant expression attempts to obtain large amounts of HBV Pol protein for structural studies have not been successful due to the insolubility of HBV Pol^7^. In fact, HBV Pol is considered to be stably folded and enzymatically active only within the nucleocapsid core particle^8^. Fortunately, the RT/RH domains of HBV Pol exhibit weak sequence homology with human immunodeficiency virus type-1 (HIV-1) RT. In particular, a consensus sequence present across multiple regions (i.e., Motifs A, B, C and D) that creates a dNTP-binding site (N-site) for HBV/HIV-1 RT is significantly conserved. Therefore, N-site structures of HBV/HIV-1 RT exhibit some degrees of similarity, though their overall structures are presumably quite different. To date, extensive X-ray structural studies of HIV-1 RT have elucidated mechanisms of reverse transcription initiation and binding/incorporation/scission of nucleoside analogue RT inhibitors (NRTIs) related to drug susceptibility/resistance^9–13^.

**Figure 1:**
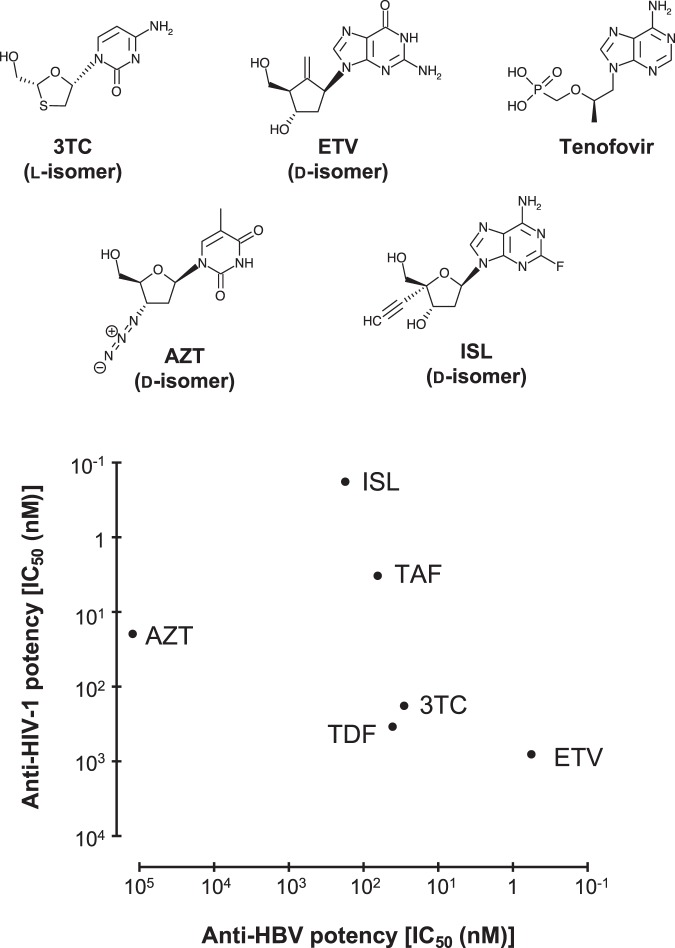
Chemical structures of NRTIs used in this study with approximate indication of their anti-HIV-1 and anti-HBV potency based on previously reported IC_50_ values^30,41^. Both TDF and TAF are prodrugs of tenofovir and are converted to tenofovir intracellularly.

Because HBV RT is an essential enzyme for viral replication, NRTIs are vital anti-HBV chemicals, and three NRTIs, lamivudine (3TC), entecavir (ETV) and tenofovir, have been widely used for anti-HBV treatment^14^. These NRTIs are tri-phosphorylated by intracellular kinases, and inhibit RT through binding to the N-site of RT. Tenofovir, an NRTI lacking a complete ribose ring, is a powerful antiviral drug for both anti-HIV-1 and anti-HBV treatment^15^. Chemical structures of typical NRTIs and their anti-HIV-1/HBV potencies are summarized in Fig. 1. Tenofovir is known to cause renal toxicity in some patients^16^. However, treatment with the recently approved tenofovir alafenamide (TAF) has significantly alleviated the renal and bone safety, is generally well tolerated, and now serves as a first-choice NRTI against HBV and HIV^15^. In addition, significant tenofovir (TDF or TAF) resistance-conferring amino acid substitutions have not been reported to date. 3TC and ETV are less toxic anti-HBV chemotherapeutics with high potency; however, drug-resistant viruses have emerged in individuals given 3TC/ETV^17^. The amino acid substitutions, M204V/I and M204V/I + L180M, in HBV RT are known to cause 3TC resistance, and these mutations significantly reduce ETV effectiveness against HBV, thereby increasing the likelihood of subsequently developing greater ETV resistance through getting additional amino acid substitutions such as S202G in HBV RT^18^. Previous characterization of HBV RT mutants suggested that the M204V/I mutation is crucial for both 3TC/ETV resistance. 3TC is also approved as an anti-HIV-1 agent, and M184V in HIV-1 RT, which corresponds to M204V in HBV RT, has also been reported as an amino acid substitution responsible for 3TC resistance in HIV-1^19^. Both 3TC and ETV are bulkier NRTI compared with tenofovir, although their structures are considerably different from each other: 3TC is a l-nucleoside analogue that has an oxathiolane instead of a ribose ring, whereas ETV is a common d-nucleoside guanosine analogue with protruded cyclopentyl methylene. It is therefore of crucial importance to understand why the key M204V/I amino acid substitution leads to both 3TC and ETV resistance.

Of note, all previous studies predicting interactions between HBV RT and NRTIs have been conducted by *in silico* modeling and docking simulation, using crystal structures of HIV-1 RTs^20–23^. However, experimental structural studies are needed to predict the N-site structure of HBV RT with higher precision. We therefore newly generated HBV RT-mimicking HIV-1 RT mutants by substituting unconserved N-site residues with corresponding amino acids in the HBV RT sequence. We recently reported that the HBV-associated Q151M mutation alone in HIV-1 RT renders HIV-1 highly sensitive to ETV, and we determined the crystal structure of HIV-1 RT^Q151M^:DNA complexed with ETV-triphosphate (ETV-TP)^24^. That structure revealed that the exocyclic methylene of ETV exerts pressure on the Met184 side-chain, pushing it backwards. This moving interaction between methylene and Met184 was not predicted by an *in silico* study^25^. Accordingly, we assumed that crystallographic studies of HIV-1 RT containing HBV-associated amino acids at the N-site should also provide important clues for understanding the mechanism of 3TC/ETV resistance caused by common M204V/I in HBV RT (M184V/I in HIV-1 RT).

Here, we report the acquisition of 3TC/ETV resistance using HIV-1 with three HBV-associated amino acid substitutions (F115Y/Y116F/Q151M) in the RT N-site. Furthermore, we determined a series of crystal structures for HIV-1 RT^F115Y/Y116F/Q151M^:DNA in complex with 3TC-triphosphate (3TC-TP) and ETV-triphosphate (ETV-TP), and with their reference natural substrates dCTP and dGTP, respectively. These structures provide a basis for understanding the common structural mechanism of the acquisition of resistance to 3TC and ETV conferred by the M184V/I and should aid in structure-based drug design to develop new agents effective against drug-resistant HBV.

## Results and Discussion

### Design of HBV-mimicking HIV-1 RT mutants

Three sequence regions form the N-site surface (Motifs A, B and C), and we newly generated an HIV-1 RT with various combinations of HBV-associated amino acid substitutions within these motifs (Table 1 and Supplementary Fig. S1). However, we found that multiple mutations, including F160L, significantly compromised viral replicability; therefore, we were unable to perform an antiviral assay to verify drug susceptibility and resistance of the mutants. Of these various HBV-mimicking HIV-1 RT mutants, we found that one with triple mutations (HIV-1 RT^F115Y/Y116F/Q151M^) was suitable for exploring the 3TC/ETV resistant mechanism, since replication-competent HIV-1^F115Y/Y116F/Q151M^ exhibits substantial 3TC/ETV susceptibility^24^, compared with HIV-1^Q151M^ and HIV-1^WT^. Furthermore, the residues 115/116/151 of HIV-1 RT are crucial targets to mimic the HBV RT N-site as these three residues are located considerably close to the ribose ring moiety of the bound dNTP/NRTI (Supplementary Fig. S1b). M184V/I in HIV-1 RT has long been known as a critical 3TC-resistant-associated mutation in HIV-1^26^. The corresponding M204V/I mutation in HBV RT has also been identified as a key mutation highly affecting HBV’s sensitivity to 3TC and ETV, and in particular, triple mutations M204V/L180M/S202G render HBV completely resistant to both 3TC and ETV^18^. Therefore, we chose three corresponding mutations, M184V, F160M, and Q182G, in HIV-1 RT to investigate resistance to 3TC/ETV in HIV-1^F115Y/Y116F/Q151M^ to 3TC and ETV.

**Table 1:** HIV-1 with HBV-associated mutations in RT created in previous and present study for investigation of typical anti-HIV-1/HBV NRTI susceptibility mechanism.

Identifier	Mutations in RT	Viral replication	References
HIV^WT^	—	++	—
HIV^Q151M^	Q151M	++	^24^
HIV ^2M^	Q151M/F160L	−	^24^
HIV^3MA^	G112S/D113A/Q151M	+	^24^
HIV^3MB^	Y115F/F116Y/Q151M	++	^24^
HIV^3MC^	I63V/L74V/Q151M	++	^24^
HIV^3MB/M184V^	Y115F/F116Y/Q151M + M184V	++	This study
HIV^3MB/F160M/M184V^	Y115F/F116Y/Q151M + F160M/M184V	++	This study
HIV^3MB/F160M/Q182G/M184V^	Y115F/F116Y/Q151M + F160M/Q182G/M184V	−	This study
HIV^6M^	G112S/D113A/Y115F/F116Y/Q151M/ F160L	−	^32^
HIV^7MA^	G112S/D113A/Y115F/F116Y/V118L/Q151M/ F160L	−	^32^
HIV^7MB^	G112S/D113A/F115Y/F116Y/L149I/ Q151M/F160L	−	^32^
HIV^7MC^	G112S/D113A/F115Y/F116Y/Q151M/I159L/F160L	−	^32^

### Comparative analysis of HBV resistance to ETV and 3TC exploiting HBV-adapted HIV-1 mutants

We prepared HIV-1 mutants with HBV-associated amino acid substitutions Q151M (HIV^Q151M^), F115Y/Y116F/Q151M (HIV^3MB^), 3MB and M184V (HIV^3MB/M184V^), 3MB and F160M/M184V (HIV^3MB/F160M/M184V^), and 3MB and F160M/Q182G/M184V (HIV^3MB/F160M/Q182G/M184V^), and examined their viral replication kinetics (Table 1). We found that HIV^3MB/F160M/Q182G/M184V^ failed to replicate, while the remaining four variants were replication-competent, suggesting that the Q182G mutation is detrimental for HIV-1 replication (Supplementary Fig. S2). The HIV-1 RT structures revealed that side-chain of Gln182 forms hydrogen-bonds with the side-chain of Arg172 and Thr165. These interactions may contribute to enzymatic stability of HIV-1 RT, and thus Q182G is unlikely to be suitable for helping understand the mechanism of HBV’s resistance to 3TC and ETV due to S202G in HBV RT. Gln182 is located approximately 10 Å away from the bound NRTI, and the structure of such a region containing Gln182 may considerably vary between HIV-1 and HBV RT than previously expected.

We next performed an antiviral assay for HIV^Q151M^, HIV^3MB^, HIV^3MB/M184V^ and HIV^3MB/F160M/M184V^, using ETV, 3TC, tenofovir alafenamide (TAF), islatravir (ISL; EFdA) and azidothymidine (AZT) (Fig. 1). The IC_50_ values of these typical NRTIs for the HIV^WT^ and variants are summarized in Table 2. We confirmed previous reports that HIV^Q151M^ and HIV^3MB^ were substantially susceptible to ETV and 3TC, but significantly less susceptible to AZT due to Q151M and F116Y mutations, which are components of the well-known multi-drug resistance Q151M-complex^24,27,28^. Importantly, we found that additional mutations F160M/M184V in RT rendered HIV^3MB^ virtually fully resistant to both 3TC and ETV (Table 2). These results strongly suggest that HBV’s resistance to 3TC and ETV (with M204V/L180M in HBV RT) is reasonably well simulated using an HIV-1 with HBV-associated 3MB mutations.

**Table 2:** Results of antiviral assay with typical anti-HIV-1/HBV NRTIs.

	IC_50_ (nM) ± SD				
NRTIs	HIV^WT^	HIV^Q151M^	HIV^3MB^	HIV^3MB/M184V^	HIV^3MB/F160M/M184V^
ETV	2605 ± 76	100 ± 11	96 ± 24	451 ± 89	1136 ± 264
3TC	363 ± 51	108 ± 6	93 ± 11	600 ± 158	1099 ± 117
TAF	3.3 ± 0.1	3.2 ± 0.3	1.6 ± 0.5	16 ± 1	54 ± 3
ISL	0.42 ± 0.2	0.17 ± 0.06	0.034 ± 0.01	2.9 ± 0.06	4.5 ± 0.3
AZT	21 ± 3	252 ± 22	985 ± 142	1148 ± 210	302 ± 57

IC_50_ (50% inhibitory concentration) values were determined by the amounts of HIV-1 p24 antigen in the culture supernatants.

ISL is known as an exceptionally powerful NRTI for HIV-1^29^. However, ISL’s potency that we observed against HIV^Q151M^ and HIV^3MB^ is not quite consistent with ISL’s moderate activity against HBV (IC_50_ = 160 nM)^30^. Previous structural studies of HIV-1 RT^WT^ in complex with ISL-triphosphate showed that a 4′-ethynyl of ISL is inserted into the hydrophobic hollow at the N-site, and Phe160 at the bottom of the hollow is likely a key amino acid to accommodate the 4′-ethynyl^31^. Because HIV^3MB/F160M/M184V^ remains susceptible to ISL, we suspect that Met160 does not interfere with the binding of 4′-ethynyl. However, in the HBV RT sequence, the corresponding residue is Leu180, although the F160L mutation renders the virus replication-incompetent, thus eliminating the possibility of measuring the effects of F160L on susceptibility to ISL. A previous X-ray structure for HIV-1 RT with HBV-associated septuple amino acid substitutions, which includes F160L, suggests that Leu160 renders the hollow shallower and may lead to a decrease in fitness mediated by 4′-ethynyl^32^. TAF is the latest tenofovir prodrug approved for anti-HIV-1 and anti-HBV treatment, and is known as a potent and robust NRTI against drug resistance^33^. Our data clearly indicate that TAF is also effective against all the HIV-1 variants we synthesized in this study (Table 2).

### X-ray structures of HIV-1 RT^3MB^:DNA in complex with 3TC-TP/ETV-TP/dCTP/dGTP

**Figure 2:**
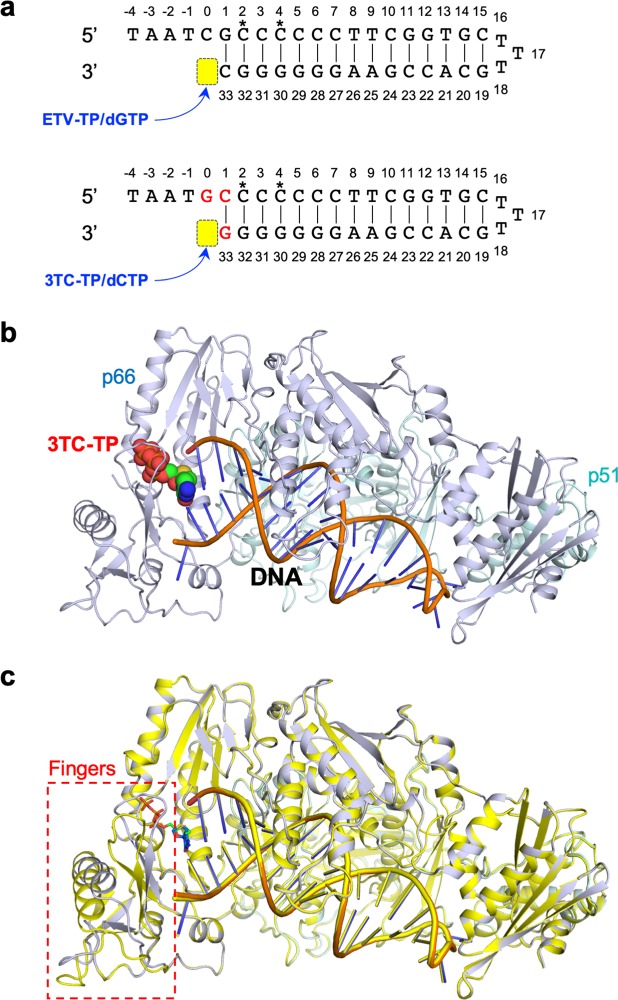
Structure analyses of HIV-1 RT^3MB^/RT^3MB/F160M/M184V^:DNA:NRTIs/dNTPs ternary complex. (**a**) Hairpin template-primer-mimicking DNA aptamer for accommodation of dGTP/ETV-TP, and newly designed aptamer with three-base substitution indicated in red for accommodation of dCTP/3TC-TP. Asterisks represent 2′-O-methyl modification. N-site positions are highlighted in yellow. (**b**) Ribbon diagram for overall structure of HIV-1 RT^3MB^:DNA:3TC-TP ternary complex. The p66 and p51 subunits are colored in light blue and cyan, respectively. The bound DNA aptamer and the 3TC-TP are shown in stick and sphere models, respectively. (**c**) Structural superimposition of HIV-1 RT^3MB^:DNA:3TC-TP and the open conformation of RT^3MB/F160M/M184V^:DNA:3TC-TP colored in yellow. DNA and 3TC-TP are shown as stick models. A conformational difference observed in the finger domain is indicated with a red dotted square.

Recombinant HIV-1 RT^3MB^ was overexpressed using *Escherichia coli*, and purified using Ni-affinity and ion-exchanging chromatography. We then used the RT assay to confirm enzymatic activity of the purified sample (Supplementary Table S1). We determined a total of four ternary complex structures for HIV-1 RT^3MB^, including RT^3MB^:DNA:3TC-TP, RT^3MB^:DNA:dCTP, RT^3MB^:DNA:ETV-TP, and RT^3MB^:DNA:dGTP, to a resolution of 2.51 Å, 2.56 Å, 2.32 Å and 2.30 Å, respectively (Table 3 and Fig. 2). A previously designed template-primer-mimicking hairpin DNA aptamer was used to accommodate ETV-TP/dGTP^24^. In addition, we designed a DNA aptamer with three base substitutions for accommodation of 3TC-TP/dCTP (Fig. 2a). The asymmetric unit contained two HIV-1 RT^3MB^ heterodimers [p66 subunit (chains A and C) and p51 subunit (chains B and D)] in complex with DNA (chains E and F) and NRTI/dNTP. A well-defined continuous electron density enabled us to construct the atomic model for residues 1–553 of the p66 subunit, and residues 5–213 and 231–427 of the p51 subunit. The internal residues 214–230 and N-terminal 20 residues including a His-tag in the p51 subunit, were disordered. In addition, the overall structures for the series of ternary complexes we report here superimpose well and have a main-chain root mean square deviation (RMSD) of ~1.1 Å. We also observed a closed conformation identical to previously reported conformation of HIV-1 RT:DNA:dNTP/NRTI ternary complexes.

**Table 3:** Crystallographic parameters and refinement statistics.

	RT^3MB^:DNA	RT^3MB^:DNA	RT^3MB^:DNA	RT^3MB^:DNA	RT^3MB/F160M/M184V^:DNA
Bound NRTI/dNTP	3TC-TP	dCTP	ETV-TP	dGTP	3TC-TP
PDB code	6KDJ	6KDK	6KDM	6KDN	6KDO
**Data collection**					
Beamline	PF BL-1A	PF BL-1A	PF BL-1A	PF BL-17A	PF BL-1A
Detector	Eiger X4M	Eiger X4M	Eiger X4M	Pilatus3S 6 M	Eiger X4M
Wavelength (Å)	1.10000	1.10000	1.10000	0.98000	1.10000
Space group	*H*3	*H*3	*H*3	*H*3	*H*3
Unit-cell parameters (Å)	*a = b = *285.1, *c = *96.0	*a = b = *284.9, *c = *95.9	*a = b = *284.4, *c = *95.8	*a = b = *284.2, *c = *95.5	*a = b = *284.2, *c = *95.9
Resolution (Å)^*^	50–2.51 (2.55–2.51)	50–2.56 (2.60–2.56)	50–2.32 (2.36–2.32)	50–2.30 (2.34–2.30)	50–2.57 (2.62–2.57)
Unique reflections	99,368	93,456	125,038	130,151	91,600
*R*_meas_^*,†^	0.105 (0.853)	0.092 (1.104)	0.098 (0.944)	0.069 (0.888)	0.078 (0.892)
Mean *I*/σ (*I*)^*^	10.6 (2.1)	13.0 (1.8)	11.6 (2.1)	17.2 (2.2)	14.5 (2.0)
Completeness (%)^*^	99.9 (100.0)	100.0 (99.4)	100.0 (100.0)	100.0 (100.0)	99.8 (96.3)
Multiplicity^*^	5.3 (5.6)	5.5 (5.5)	5.4 (5.6)	5.2 (5.1)	5.4 (5.1)
Wilson *B*-factor (Å^2^)	55.1	55.9	44.1	45.3	60.5
**Refinement**					
*R*_work_/*R*_free_^‡,§^	0.182/0.230	0.190/0.234	0.190/0.223	0.186/0.221	0.192/0.230
No. of atoms	17,551	17,528	17,780	17,721	17,434
Average *B*-factors (Å^2^)					
All/DNA	68.9/68.2	70.2/70.3	59.0/55.9	59.3/57.1	75.3/75.3
Ligand	88.5 (3TC-TP)	63.6 (dCTP)	62.0 (ETV-TP)	54.3 (dGTP)	111.5 (3TC-TP)
R.m.s.d. from ideal					
Bond lengths (Å)	0.009	0.004	0.005	0.005	0.005
Bond angles (°)	1.046	0.805	0.794	0.722	0.909
Ramachandran plot^¶^					
Favored/Outliers (%)	95.86/0.05	96.27/0.16	96.54/0.21	95.86/0.16	96.80/0.16

^*^Values in parentheses are for the outermost resolution shell.

^†^*R*_meas_ = Σ_h_ Σ_*i*_ |*I*_h,*i*_ − <*I*_h_>|/Σ_h_Σ_*i*_ *I*_h,*i*_, where <*I*_h_> is the mean intensity of a set of equivalent reflections.

^‡^*R*_work_ = Σ |*F*_obs_ – *F*_calc_|/Σ *F*_obs_ for 95% of the reflection data used in the refinement. *F*_obs_ and *F*_calc_ are the observed and calculated structure factor amplitudes, respectively.

^§^*R*_free_ is the equivalent of *R*_work_, except that it was calculated for a randomly chosen 5% test set excluded from refinement.

^¶^Ramachandran analysis was performed using the program MolProbity^54^.

### N-site structure of the HIV-1 RT^3MB^:DNA:NRTI/dNTP ternary complex

**Figure 3:**
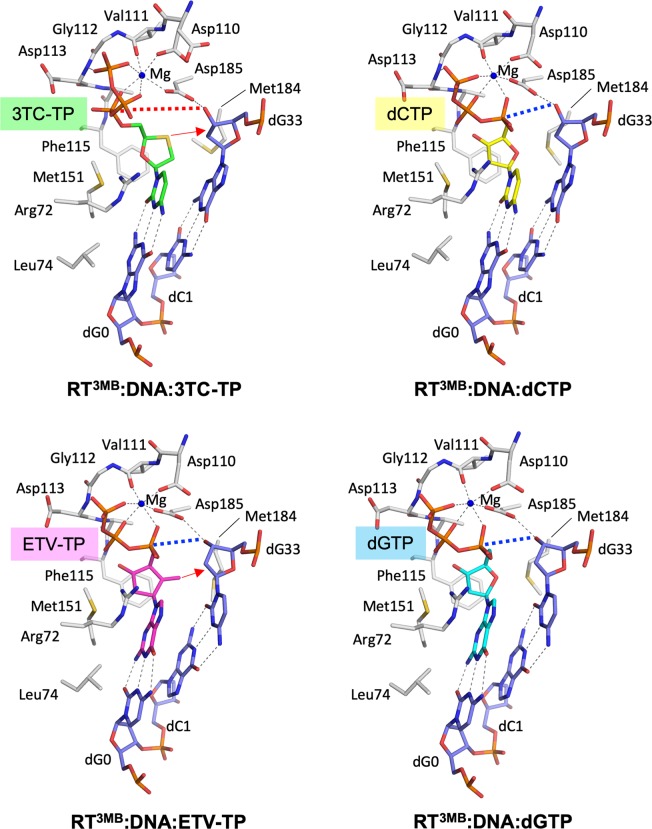
N-site structures of the HIV-1 RT^3MB^:DNA in complex with 3TC-TP, dCTP, ETV-TP and dGTP. Carbon atoms for the RT amino acid side-chain creating N-site are colored white, while nitrogen, oxygen, phosphorous and sulfur atoms are colored in blue, red, orange and yellow, respectively. Carbon atoms for the 3TC-TP/ETV-TP/dCTP/dGTP are colored in green, magenta, yellow and cyan, respectively. Carbon atoms for the DNA aptamer are colored in light blue. The pushing effect of oxathiolane/exocyclic methylene against the Met184 side chain is depicted with red arrows. Hydrogen bonds and metal-chelating interactions are drawn with gray thin dotted lines. The distance between the α-phosphate of 3TC-TP and 3′-end hydroxyl of DNA aptamer (~7.1 Å) is shown with the red dotted line. The corresponding distances in d-isomer complexes (ranging 4–5 Å; Supplementary Table S2) are also shown with blue dotted lines.

We have elucidated the structure for trapped 3TC-TP/ETV-TP/dCTP/dGTP at the N-site of HIV-1 RT^3MB^:DNA (Fig. 3). The electron density for bound DNA, NRTI/dNTP and N-site residues was unambiguous (Supplementary Figs. S3 and S4), thus elucidating detailed interatomic interactions between RT^3MB^, DNA and NRTI/dNTP. In addition, single Mg^2+^ bound at the N-site in a typical octahedral coordination with three oxygen atoms derived from the triphosphate moiety of NRTI/dNTP, two carboxylic oxygens of Asp110 and Asp185, and the main-chain carbonyl oxygen of Val111 (Fig. 3). We noted that the side-chain of Asp110 (chain A) in RT^3MB^:DNA:3TC-TP was flipped and did not coordinate with Mg^2+^. As a result, the refined *B*-factor value for the Mg^2+^ (chain A) in RT^3MB^:DNA:3TC-TP was relatively high (~100 Å^2^). Nevertheless, the simulated annealing Fo-Fc omit map indicates reliable binding of Mg^2+^ at the N-site in all ternary complexes determined in this study (Supplementary Fig. S4), and the *B*-factors of all Mg^2+^ have been reasonably refined with full occupancy and a lack of any negative Fo-Fc peaks at the N-site.

### Atypical binding conformation of 3TC-TP to HIV-1 RT^3MB^:DNA

**Figure 4:**
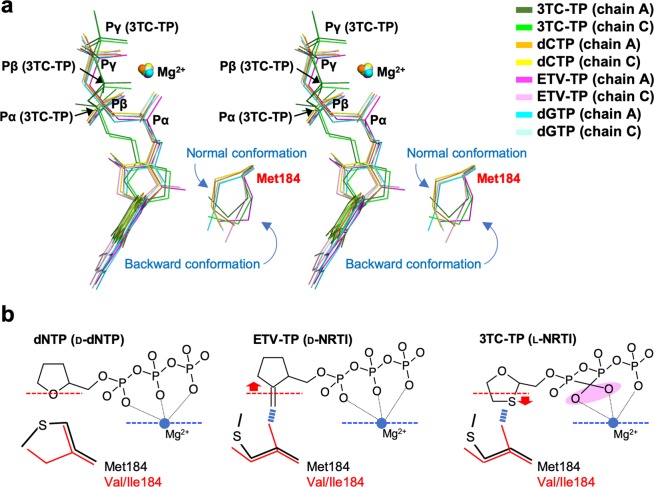
Positional and conformational differences among the bound dNTP, ETV-TP and 3TC-TP. (**a**) Stereo-view superimposition of the bound 3TC-TP (chain A, dark green; chain C, light green), dCTP (chain A, orange; yellow, chain C), ETV-TP (magenta, chain A; light pink, chain C), and dGTP (cyan, chain A; light cyan, chain C) in HIV-1 RT^3MB^ analyzed in this study. The α-, β-, γ-phosphorous atoms of the NRTIs/dNTPs are indicated. The bound Mg^2+^ and Met184 side-chains in each chain are also shown, and the bimodal conformations of Met184 are indicated as normal and backward conformations (see text). (**b**) Schematic diagram showing relative location and conformation of triphosphates of dNTP, ETV-TP and 3TC-TP. Position of ribose oxygen of the dNTP and Mg^2+^ are depicted by dotted lines in red and blue, respectively. The expected steric hindrance between Cγ of Val/Ile184 and the methylene of ETV/oxathiolane of 3TC are shown by thick dotted lines in blue. Unique spatial replacement of α- and β-phosphate oxygens of 3TC-TP is highlighted in pink.

Our ternary complex structures reveal that 3TC-TP binds at the N-site with its triphosphate moiety in an atypical conformation compared to the natural d-isomer form of NRTIs/dNTPs (Figs. 3 and 4). The α-phosphate of the d-isomers lies closer to the 3′-hydroxyl of the primer DNA, whereas that of 3TC-TP occupies the position farther from the primer 3′-hydroxyl, and is instead closer to main-chain Ala114-Phe115 residues (Fig. 3). The conformational difference of triphosphate between l/d-isomers leads to the spatial replacement of α- and β-phosphate oxygens coordinated to Mg^2+^. Despite this significantly different spatial arrangement of triphosphate, the Mg^2+^ occupies an identical position, retaining an identical octahedral coordination (Figs. 3 and 4). Recently, Bertoletti *et al*. have reported a crystal structure for wild-type HIV-1 RT with bound 3TC-TP (PDB code, 6OUN), which also revealed a unique binding conformation of 3TC-TP, roughly similar to our structure^34^, although with two significant structural differences. First, planar base-pairing does not occur for dG705:3TC-TP in the structure of 6OUN, and the angle of tilt was approximately 22° (Supplementary Fig. S5). In contrast, our structure displays nearly planar base pairing for the corresponding dG0:3TC-TP. Second, the bridging oxygen between Pα and Pβ is coordinated with Mg^2+^ in the structure of 6OUN, instead of the α-phosphate oxygen coordination that we observed in our structure. These differences imply that bound 3TC-TP in the present study binds more tightly to three non-bridging phosphate oxygen atoms that act as Mg^2+^ coordinate ligands. This binding pattern has also been observed in all reported RT structures complexed with d-isomer NRTIs/dNTPs. It is likely that the slightly skewed binding mode of 3TC-TP in the RT^WT^ may be unstable, resulting in a prerequisite conformational state of the 3TC-TP at the N-site. In both structures, the distance between the primer 3′-hydroxyl and the α-phosphate was farther (~7 Å) compared with that observed for the d-isomer complex (~5 Å) (Supplementary Table S2), which explains the slow 3TC incorporation rate reported in previous *in vitro* experiments^35,36^. In addition, another cell-based study indicated the 3TC might not be incorporated to DNA differently from the AZT, a known polymerase chain terminator^37^. The bound 3TC-TP conformation clearly indicates recalcitrance towards incorporation into DNA, and thus 3TC appears to be an N-site blocker hindering the binding of substrate dCTP rather than a chain terminator^38^.

### Oxathiolane/methylene of 3TC-TP/ETV-TP directly moves the Met184 (Met204 in HBV RT) side chain backward

The HIV-1 RT^3MB^:DNA:3TC-TP ternary complex revealed that the thioether moiety of 3TC-TP oxathiolane lies closer to the Met184 and ribose ring of the 3′-end nucleotide, and consequently the side-chain of the Met184 is moved backward, as in the conformation of Met184 observed in the RT^3MB^:DNA:ETV-TP complex (Figs. 3 and 4). Electron density for all Met184 side-chains analyzed in this study was well-defined (Supplementary Fig. S6), and structural superimposition revealed that the conformation of the Met184 side-chain could be classified into two conformational modes: a “normal conformation” located near the ribose ring of the bound dGTP/dCTP with appropriate van der Waals distances, and a “backward conformation” pushed backward by the exocyclic methylene/oxathiolane ring of ETV-TP/3TC-TP (Fig. 4). Based on the present structures, we suspect that substitution of Met184 with Val/Ile would cause steric clash between a Cγ atom of Val/Ile and the methylene/thioether of ETV/3TC, assuming the Cγ atoms of Val/Ile are positioned similarly to those in the two conformations of Met184 (Fig. 4b). A possible steric clash has been predicted in previous *in silico* modeling studies^20–23^, but the oxathiolane orientation toward Met184 with an atypical triphosphate conformation had not been predicted. It is also noteworthy that in a recently reported experimental structure for the RT^WT^:DNA:3TC-TP complex, the orientation of oxathiolane to the Met184 was not observed, and the side-chain of Met184 retained normal conformation with a 3.8 Å distance between the sulfur atom of 3TC-TP and Cγ (Supplementary Table S2 and Fig. S5c)^34^. As described above, the RT^WT^:DNA:3TC-TP complex structure revealed slightly skewed and loose binding of 3TC-TP with Met184 in normal conformation. Thus, a M184V/I steric clash would likely occur when 3TC-TP tightly binds to the N-site, as observed in this study. Previous antiviral assays demonstrated that the IC_50_ value of 3TC against HBV was approximately ten-fold lower than that against HIV-1^30^. Our data also demonstrated that HIV^3MB^ is approximately 4-fold more sensitive to 3TC than HIV^WT^ (Table 2). Tight binding of 3TC-TP observed at the N-site of RT^3MB^ may explain this result. A structural comparison also indicates that HBV-associated bulky Met151 and the slightly shifted phenyl ring of HBV-associated Phe115 might contribute to the adhesive approach of 3TC-TP toward the Met184 side-chain in RT^3MB^ (Supplementary Fig. S7).

### Bound 3TC-TP is misaligned in the N-site of RT^3MB/F160M/M184V^ due to Val184 Cγ1

**Figure 5:**
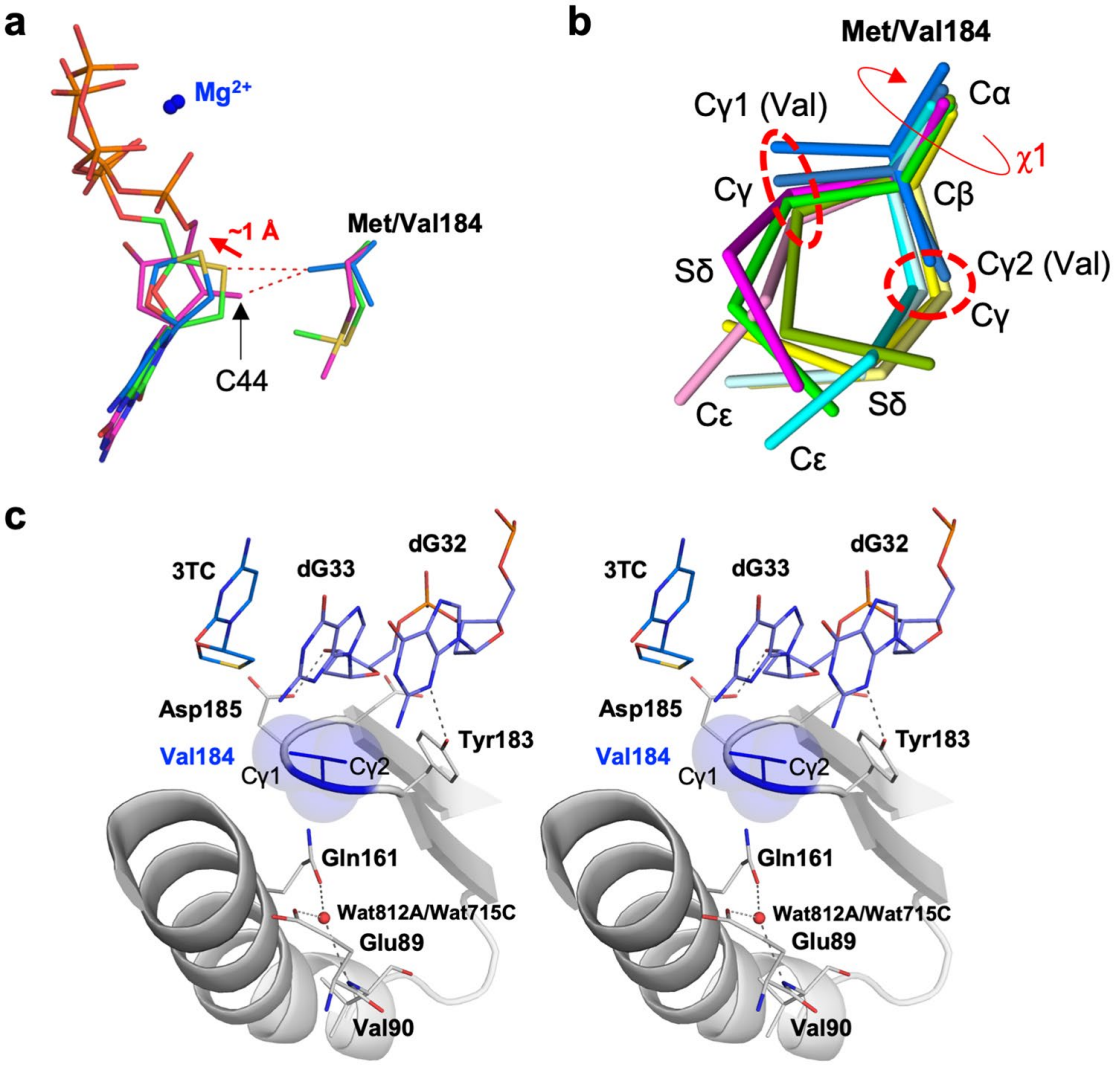
N-site structure of RT^3MB/F160M/M184V^:DNA:3TC-TP ternary complex. (**a**) Superimposition of 3TC-TP in RT^3MB^, ETV-TP in RT^3MB^, and modeled 3TC moiety of 3TC-TP in RT^3MB/F160M/M184V^ representing the deviation by ~1 Å of 3TC moiety in RT^3MB/F160M/M184V^, as indicated by red arrow. Side-chains of Met/Val184 are also shown. Carbon atoms for 3TC-TP in RT^3MB^, ETV-TP in RT^3MB^, and 3TC moiety in RT^3MB/F160M/M184V^ are colored in green, magenta and dark blue, respectively. C44 atom of ETV-TP (a tip of the methylene group) is also labeled. (**b**) Superimposition of all of the Met/Val184 side-chains analyzed in this study. The Cγ1 and Cγ2 atoms of Val184 coincide with the positions of Cγ atom in the normal and backward conformations of Met184, respectively, as indicated by red dotted circles. Each atom is labeled, and the χ1 rotation about the Cα-Cβ bond is also indicated by red arrow. (**c**) Stereo-view structure of Val184 and nearby residues in the RT^3MB/F160M/M184V^:DNA:3TC-TP complex. Val184 side-chain is represented by sphere model and the Cγ1 and Cγ2 atoms are labeled. The residues, solvent and DNA aptamer nucleotides near the Val184 are shown with a stick model, and the hydrogen bonding interactions formed within those residues are shown with dotted lines.

To experimentally explore the structural effects of the Met184 substitution with Val/Ile, we also determined the HIV-1 RT^3MB/F160M/M184V^:DNA:3TC-TP ternary complex structure at a resolution of 2.57 Å (Table 3). We found that two RT molecules in the asymmetric unit were in different conformations: one in a closed conformation identical to other RT^3MB^ ternary complexes, whereas the other molecule was in an open conformation reported previously for the RT^Q151M^:DNA binary complex structure (Fig. 2c)^24^. Despite the difference in RT conformations, a well-defined electron density observed at both RT N-sites enabled us to build the atomic model for the cytosine base and oxathiolane moiety of 3TC-TP. However, electron density for the triphosphate and Mg^2+^ was quite ambiguous and we were unable to construct the model (Supplementary Fig. S4), indicating loose binding of 3TC-TP to RT^3MB/F160M/M184V^. The structure revealed that oxathiolane deviates from that in the RT^3MB^ ternary complex by a maximum deviation of ~1 Å (Fig. 5a). The sulfur atom is located at the gap between Asp185 side-chain carboxyl and the 3′-end nucleotide ribose of the primer, with a distance between the sulfur atom of 3TC-TP and Cγ1 of Val184 in chains A and C of 4.37 Å (chain A) and 4.06 Å (chain C), respectively (Supplementary Table S2). The structural superimposition of RT^3MB/F160M/M184V^ and RT^3MB^ also showed that both the methylene C44 of ETV-TP and the sulfur of 3TC-TP are located 2.8–3.0 Å from Cγ1 of Val184, indicating a severe steric clash (Fig. 5a). The side-chain structure of Val and Ile is the same but Ile has just one methyl group greater than Val. Thus, a nearly identical ETV/3TC resistance mechanism appears to work for resistance to ETV and 3TC with M184I (Fig. 4b). Interestingly, the Cγ1 and Cγ2 of Val184 coincides with Cγ of Met184 in normal and backward conformations, respectively, indicating that the χ1 angle of the residue 184 side-chain is significantly restricted (Fig. 5b). Residue 184 is sandwiched by a rigid primer chain (dG32-dG33) and Gln161 forming hydrogen-bonds with solvent, Glu89 side-chain, and the main-chain carbonyl of Val90 (Fig. 5c). These elaborate interatomic interactions nearby residue 184 well explain why the observed conformation of the Val184 side chain is the only possible one where its Cγ atoms allow conformational bimodality of the Met184 side chain.

### Implications for drug design to overcome the resistance acquisition of HIV-1 and HBV against ETV, 3TC, and other NRTIs

The present structure analyses suggest that the interactions of the Met184 side-chain with oxathiolane of 3TC-TP and methylene of ETV-TP are likely responsible for tight binding of 3TC-TP and ETV-TP at the N-site, respectively. Therefore, the branched chain of Val and Ile is expected to be incompatible with the binding of 3TC-TP and ETV-TP to the N-site. One possible solution to overcome M184V/I (M204V/I in HBV) resistance is to decrease composition for NRTIs that do not rely on the interactions with Met184. Tenofovir is a compound with a slim structure lacking a complete ribose ring. Previously reported X-ray structure of HIV-1 RT^WT^ complexed with tenofovir-diphosphate revealed that the acyclic linker connecting base with phosphate moieties minimally interacts with N-site residues and lies distant from Met184^12,39^. In fact, M184V in HIV-1 (or M204V in HBV) is not reportedly associated with TDF/TAF resistance^40^. In the present study, we showed that the susceptibility of HIV-1 carrying mutant RTs with M184V (HIV-1^3MB/M184V^ and HIV^3MB/F160M/M184V^) to TAF was substantially reduced (Table 2). Thus, it is apparent that the HIV-1 RT mutants with ETV-associated drug resistant mutation (M184V) presented in this study may not precisely reproduce the binding mode of TDF/TAF to HIV-1 RT with M184V, suggesting the imperfection of the present HBV RT model. Further experiments with more amino-acid substitutions introduced around the N-site should enable us to generate a more accurate HBV RT model(s) to study HBV drug-resistance to ETV and other NRTIs including TDF/TAF. Another possible solution is to extend the interactions between NRTI and RT to compensate for the steric conflict at Val/Ile184. We have previously reported that certain NRTIs with 4′-modification demonstrate strong potency against HIV-1/HBV, and in particular, 4′-cyano-containing NRTIs were found to be potent against both HIV-1 and HBV^30,41^. Although the structure of RT with bound 4′-cyano-containing NRTI-TP has not yet been determined, we suspect that the 4′-cyano most likely exploits the hydrophobic pocket surrounded by Ala114, Phe115, Phe160 and Met184, and forms extended interactions with these hydrophobic residues. Previous *in vitro* antiviral assays have demonstrated that 4′-cyano-containing NRTIs, such as CAdA and CdG, show slightly decreased but moderate activity against HBV with M204V in RT^30^. In addition, hydrophilic modification of exocyclic methylene may also utilize interactions with the main-chain amide of Asp185, side-chain carboxyl of Asp185, or 3′-hydroxyl of the primer, to stabilize the binding of modified NRTI at the N-site. Combined with the aforementioned strategies, such modifications could pave the way for overcoming the critical drug resistance of HIV-1 and HBV.

Notably, anti-HBV therapy with an NRTI has a limitation related to its efficacy. Currently available anti-HBV NRTIs including TAF develop adverse events and not completely efficacious in some individuals with HBV. Furthermore, monotherapy with NRTI(s) cannot eliminate HBV because covalently-closed-circular DNA (cccDNA) of HBV in the cytoplasm of liver cells exists even with prolonged antiviral therapy. Therefore, novel anti-HBV strategies directed against other steps of the viral replication cycle, in combination with current antiviral therapy with an NRTI(s), should be developed to achieve a functional cure for HBV infection^42^.

## Conclusion

We generated a 3TC/ETV-sensitive HIV-1 variant with HBV-associated mutations 3MB (F115Y/Y116F/Q151M) in RT, and successfully simulated 3TC/ETV resistance in HBV using HIV^3MB^ with mutations F160M/M184V. In addition, we determined X-ray structures of HIV-1 RT^3MB^:DNA in complex with 3TC-TP/ETV-TP/dCTP/dGTP. These structures together with a previous RT^Q151M^ in complex with ETV-TP revealed that 1) 3TC-TP binds in an atypical binding conformation dissimilar to those of common d-isomer NRTIs/dNTPs and 2) both oxathiolane of 3TC-TP and cyclopentyl methylene of ETV-TP bind through directly pushing back the Met184 side-chain. The structure of HIV-1 RT^3MB/F160M/M184V^:DNA in complex with 3TC-TP also showed that bound 3TC-TP deviated from the tight binding position observed in RT^3MB^ with disordered triphosphate moiety, indicating a steric clash evasion with Val184 Cγ1. Our structures also explain a common mechanism for 3TC/ETV resistance by severe steric clash between the M184V/I (M204V/I in HBV) side-chain and the oxathiolane/methylene of bound 3TC-TP/ETV-TP. We believe the structures reported here will serve as a model for *in silico* studies with other NRTIs and will contribute to the rational design of new agents to invalidate drug resistant mutations. Further studies should be aimed at generating more accurate HBV RT models by employing different combinations of amino acid substitutions at the HIV-1 RT N-site.

## Methods

### Preparation of recombinant HIV-1 RT mutants

HIV-1 RT expression vectors (pET28_His_6_-p51 and pCDF_p66) were previously described^24,43^. All site-specific mutations were introduced by inverse PCR mutagenesis method^44^, using the pCDF_p66 as template DNA. The mature p66-p51 heterodimeric HIV-1 RTs (HIV-1 RT^WT^, RT^Q151M^, RT^3MB^, RT^3MB/M184V^, and RT^3MB/F160M/M184V^) were produced using *E*. *coli* BL21-CodonPlus(DE3)-RIL, and purified by Ni-affinity chromatography, following multiple steps of ion-exchanging chromatography, according to a previously described procedure^24,32^. Fractions containing roughly equal molar concentrations of p66 and p51 were collected, and dialyzed against buffer containing 10 mM Tris-HCl at pH 8.0 and 100 mM NaCl. Quantification of total purified proteins was determined using Bradford protein assay (Bio-Rad) and by measuring absorbance at 280 nm with an extinction coefficient of 260,120 M^−1^·cm^−1^. The purified sample was concentrated to ~16 mg/mL using 50 kDa Amicon Ultra centrifugal filtration devices (Millipore). A small amount of concentrated sample (~ 50 μL) was diluted two-fold with buffer containing 10 mM Tris-HCl pH 8.0 and 60% glycerol, and then stored at −22 °C until use in the enzyme reverse transcriptase assay. Enzymatic activities of the purified HIV-1 RTs were assessed using Reverse Transcriptase Assay kit (Roche), according to the manufacturer’s instructions. The remaining samples were used for DNA aptamer complex formation and crystallization as described below.

### Viral replication kinetics and antiviral assay

HIV-1 wild-type NL4-3 (HIV^WT^) infectious clones containing RT mutations were constructed with the In-Fusion HD Cloning Kit (Clontech), as previously described^24^. Viral replication kinetics were measured as previously described^45^ with minor modifications. In brief, each virus variant was harvested by transfection of HIV-1 plasmids into 293 T cells using Attractene transfection reagent (QIAGEN). MT-4 cells (5 × 10^4^ cells/mL) were exposed to 200 TCID_50_ (50% tissue culture infectious dose) of each HIV-1 variant and cultured for 8 days. Quantities of HIV-1 p24 antigen were determined on days 0, 2, 4, 6, and 8 using Lumipulse HIV-1 p24 (Fujirebio). Antiviral assays using HIV-1^WT^ and replication-competent HIV-1 variants (HIV^Q151M^, HIV^3MB^, HIV^3MB/M184V^, and HIV^3MB/F160M/M184V^) were also conducted, as previously described^44^. Briefly, MT-4 cells (5 × 10^4^ cells/mL) were exposed to each virus at 200 TCID_50_ with various concentrations of antiviral agents. After 7 days, the 50% inhibitory concentration (IC_50_) was determined by measuring the amount of HIV-1 p24 antigen in culture supernatants. All assays were performed in duplicate or triplicate.

### Preparation of RT:DNA complexes and gel-filtration chromatography

Template-primer-mimicking hairpin DNA aptamers (Fig. 2a)^46,47^ were obtained from Hokkaido System Science, Co., Ltd (Sapporo, Japan). Aptamers were dissolved at 100 μM in buffer containing 10 mM Tris-HCl pH 8.0 and 1 mM EDTA. The aptamer solutions were heated at 80 °C for 10 min, and then cooled slowly to 20 °C at a cooling rate of 1 °C/min. The heat-treated aptamer was immediately mixed with purified RT sample at a molar ratio of approximately 1.2:1.0, and incubated overnight at 4 °C. The resultant RT^3MB^:DNA and RT^3MB/F160M/M184V^:DNA binary complexes were loaded on HiLoad 16/600 Superdex 200 pg gel-filtration column (GE Healthcare) with buffer containing 10 mM Tris-HCl pH 8.0 and 50 mM NaCl. Native and SDS PAGE analyses were conducted to check the content and purity of each fraction, according to a previously described method^24^. The peak fractions containing p66-p51 heterodimer with bound DNA aptamer were collected and concentrated to 20 mg/mL for crystallization experiments.

### Crystallization

Crystals of HIV-1 RT^3MB^:DNA and RT^3MB/F160M/M184V^:DNA binary complexes were obtained by the hanging-drop vapor diffusion method at 20 °C with 24-well crystallization plates. The crystallization droplets were prepared by mixing 0.5 μL sample solution and 0.5 μL reservoir solution, and then equilibrated against 500 μL of reservoir solution. The crystals were grown under heavy precipitation conditions using the reservoir solution containing 20 mM bis-Tris-HCl pH 6.0, 20–60 mM di-ammonium hydrogen citrate, 20 mM MgCl_2_, 1.5–3.5% polyethylene glycol (PEG) 6000, 2.0–2.4% sucrose and 2.4–4.8% glycerol. The single crystals were briefly soaked in cryoprotectant solution consisting of 10 mM Tris-HCl pH 8.0, 20 mM bis-Tris HCl pH 6.0, 50 mM NaCl, 20 mM MgCl_2_, 20–60 mM di-ammonium hydrogen citrate, 10% PEG 6000, 4.8% sucrose, and 25.6% glycerol, and further transferred into the same cryoprotectant solution supplemented with 2.5 mM 3TC-TP/ETV-TP/dCTP/dGTP. 3TC-TP was purchased from Abcam (Cambridge, UK). ETV-TP was prepared, as in the previously described procedure^24^. The dCTP/dGTP was obtained from Takara Bio Inc. (Shiga, Japan). The crystals were flash-cooled and stored in liquid nitrogen prior to subsequent X-ray diffraction studies.

### Structure determination and model refinement

All X-ray diffraction data reported in this study were collected using synchrotron radiation at Photon Factory BL-1A/BL-17A (PF; Tsukuba, Japan). The diffraction spots were indexed, integrated and merged using the programs XDS^48^ and AIMLESS^49^. Crystals were found to belong to space group *H*3 with unit-cell dimensions a = b = 284 and c = 98 Å. The model refinement was performed with the program REFMAC5^50^ in the CCP4 program package^51^, using the atomic model of RT^Q151M^:DNA:ETV-TP ternary complex (PDB code, 5XNL), excluding solvents and ligands as a starting model. The model corrections and fitting were conducted using the program Coot^52^. The TLS parameters were calculated and applied in the final stage of model refinement with the program Phenix^53^. The final model assessment was performed using the program MolProbity^54^. Crystallographic parameters and model refinement statistics are provided in Table 3. All molecular graphics were prepared using the program PyMol ver. 2.3.4 (Schrödinger LLC).

## Supplementary information

Supplementary information.

## Data Availability

The atomic coordinates and structure factor amplitudes of the HIV-1 RT^3MB^:DNA:3TC-TP, RT^3MB^:DNA:dCTP, RT^3MB^:DNA:ETV-TP, RT^3MB^:DNA:dGTP and RT^3MB/F160M/M184V^:DNA:3TC-TP complexes have been deposited in the RCSB Protein Data Bank under accession codes 6KDJ, 6KDK, 6KDM, 6KDN, and 6KDO, respectively. Other data generated and/or analyzed during this study are available from the corresponding authors upon reasonable request.
