# Supplementary material for: Structural features in common of HBV and HIV-1 resistance against chirally-distinct nucleoside analogues entecavir and lamivudine
Source: Sci Rep. 2020 Feb 20;10:3021. doi: 10.1038/s41598-020-59775-w (PMC7033138; doi:10.1038/s41598-020-59775-w)
Supplement: Supplementary file 1 — Supplementary information. [file 41598_2020_59775_MOESM1_ESM.pdf]

Supplementary information

Structural features in common of HBV and HIV-1 resistance against chirally-distinct nucleoside analogues entecavir and lamivudine

Yoshiaki Yasutake[1,2,*], Shin-ichiro Hattori[3], Noriko Tamura[1], Kouki Matsuda[3], Satoru Kohgo[3,4], Kenji Maeda[3*], Hiroaki Mitsuya[3,5,6]

[1]Bioproduction Research Institute, National Institute of Advanced Industrial Science and Technology (AIST), Sapporo 062-8517, Japan.
[2]Computational Bio Big-Data Open Innovation Laboratory (CBBD-OIL), AIST, Tokyo 169-8555, Japan.
[3]National Center for Global Health and Medicine Research Institute, Tokyo 162-8655, Japan.
[4]Faculty of Pharmaceutical Sciences, Sojo University, Kumamoto 860-0082, Japan.
[5]Experimental Retrovirology Section, HIV and AIDS Malignancy Branch, National Cancer Institute, National Institutes of Health, Bethesda, MD 20892, USA.
[6]Department of Clinical Sciences, Kumamoto University Hospital, Kumamoto 860-8556, Japan.

*Correspondence should be addressed to Yoshiaki Yasutake. Tel: +81-11-857-8514; Fax: +81-11-857-8980; E-mail: y-yasutake@aist.go.jp. Correspondence may also be addressed to Kenji Maeda. Tel: +81-3-3202-7181; Fax: +81-3-3207-1038; E-mail: kmaeda@ri.ncgm.go.jp.

Supplementary Table S1. Results of the enzyme assay for recombinant HIV-1 RT mutants used in the crystallographic study.

	Enzyme activity (mU/ng)	Relative activity	References
RT^{WT}	7.97 ± 0.13	1.00	This study.
RT^{3MB}	7.50 ± 0.25	0.94	
$RT^{3MB/F160M/M184V}$	0.39 ± 0.021	0.049	
RT^{WT}	6.23*	-	Yasutake *et al.*,
RT^{Q151M}	6.16*	-	2018[24]

*The enzyme activities of recombinant HIV-1 RT^{WT} and RT^{Q151M} reported in our previous study are also provided for comparison. The assays were performed in duplicate.

Supplementary Table S2. Key inter-atomic distances for the structures reported in this study.

Atom definitions	Distances (Å)		PDB code	References
	Chain A	Chain C		
3TC-TP Pα − 3′-end OH	7.12	7.10	6KDJ	This study
dCTP Pα − 3′-end OH	4.10	4.38	6KDK	
ETV-TP Pα − 3′-end OH	4.71	4.76	6KDM	
dGTP Pα − 3′-end OH	4.78	4.96	6KDN	
3TC-TP S3′ – Met184 Cβ	4.25	4.54	6KDJ	This study
3TC-TP S3′– Met184 Cγ	4.93	5.01	6KDJ	
dCTP O4′ – Met184 Cβ	4.77	4.99	6KDK	
dCTP O4′ – Met184 Cγ	3.53	3.69	6KDK	
ETV-TP C6′ – Met184 Cβ	4.86	5.07	6KDM	
ETV-TP C6′ – Met184 Cγ	5.21	5.17	6KDM	
dGTP O4′ – Met184 Cβ	4.74	4.86	6KDN	
dGTP O4′ – Met184 Cγ	3.40	3.53	6KDN	
3TC-TP S3′ – Val184 Cβ	5.40	5.33	6KDO	
3TC-TP S3′ – Val184 Cγ1	4.37	4.06	6KDO	
3TC-TP S3′ – Met184 Cβ	5.24	-	6OUN	Bertoletti *et al.*, 2019[34]
3TC-TP S3′– Met184 Cγ	3.79	-	6OUN	

a

Motif A	HIV-1 RT	109 LDVGDAYFSV 118	
	HBV RT	82 LDVSAAFYHL 91	
Motif B	HIV-1 RT	149 LPQGWKGSPAIF 160	
	HBV RT	169 IPMGVGLSPFLL 180	
Motif C	HIV-1 RT	180 IYQYMDDLYVG 191	
	HBV RT	200 AFSYMDDVVLG 211	

b

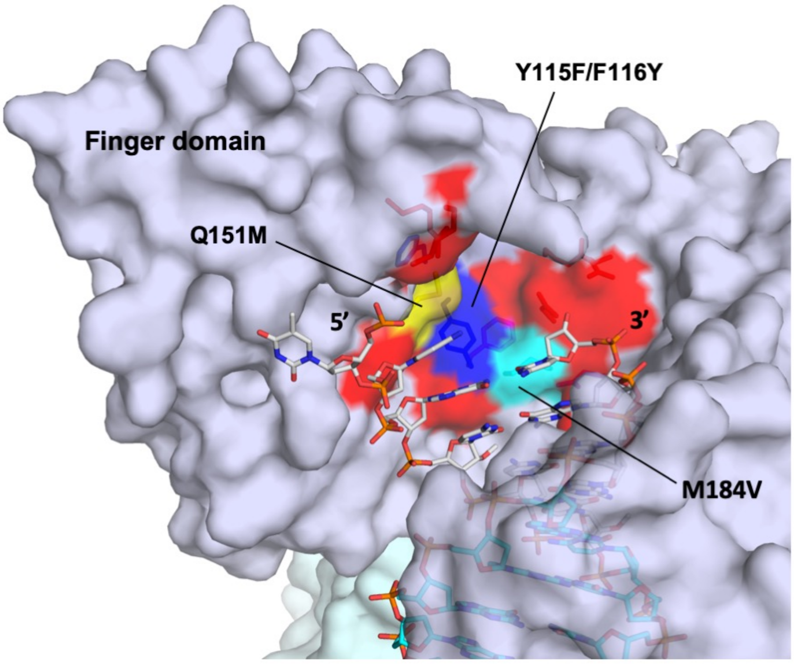

Supplementary Fig. S1. Conserved amino-acid residues between HIV-1 and HBV RT N-site. (a) Amino-acid sequence alignment between HIV-1 and HBV RT within consensus sequence Motifs A, B and C. The identical residues are colored in red. The residues for mutational analysis in this study are the following: Gln151, Tyr115/Phe116, Met184, Fhe160L/Gln182, which are highlighted in yellow, blue, cyan, and pink, respectively. (b) The molecular surface representation for the HIV-1 RT N-site. This model was generated using the previously reported RT^{Q151M}:DNA binary complex (PDB code, 5XN0)[24]. The DNA aptamer is shown with a stick model. The N-site surface is colored according to the same scheme in (a).

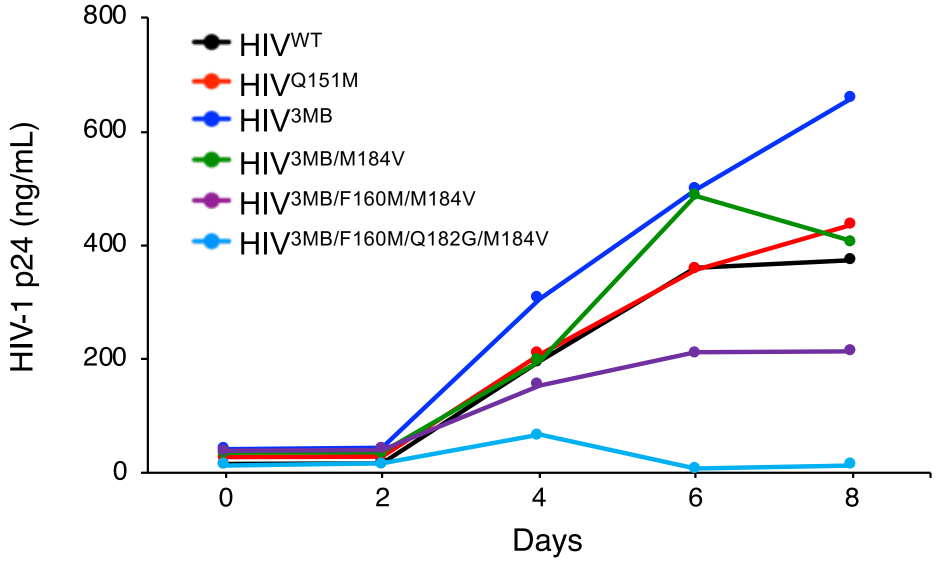

Supplementary Fig. S2. Replication kinetics of HIV-1^{WT} and the HIV-1 variants (HIV^{Q151M}, HIV^{3MB}, HIV$^{3MB/M184V}$, HIV$^{3MB/F160M/M184V}$, and HIV$^{3MB/F160M/Q182G/M184V}$). The HIV-1 p24 amounts in culture supernatants of each HIV-1 variant were determined on days 0, 2, 4, 6, and 8.

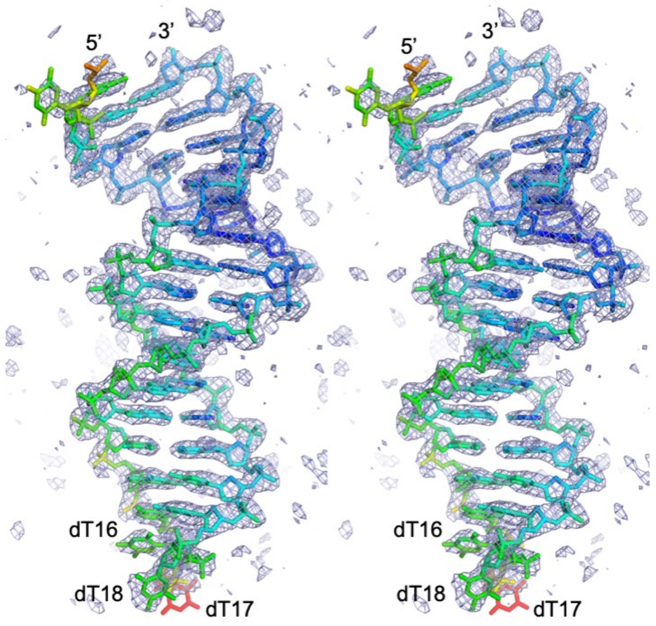

Supplementary Fig. S3. Simulated annealing $F_o - F_c$ omit map for the bound DNA aptamer in HIV-1 RT^{3MB}:DNA:3TC-TP shown in stereo-view. The DNA model is colored by *B*-factor values in the order of increasing *B*-factors from blue to red. Three dTs (dT16-dT18) forming hairpin loop of the aptamer are indicated. The map is contoured at a 3.0σ level.

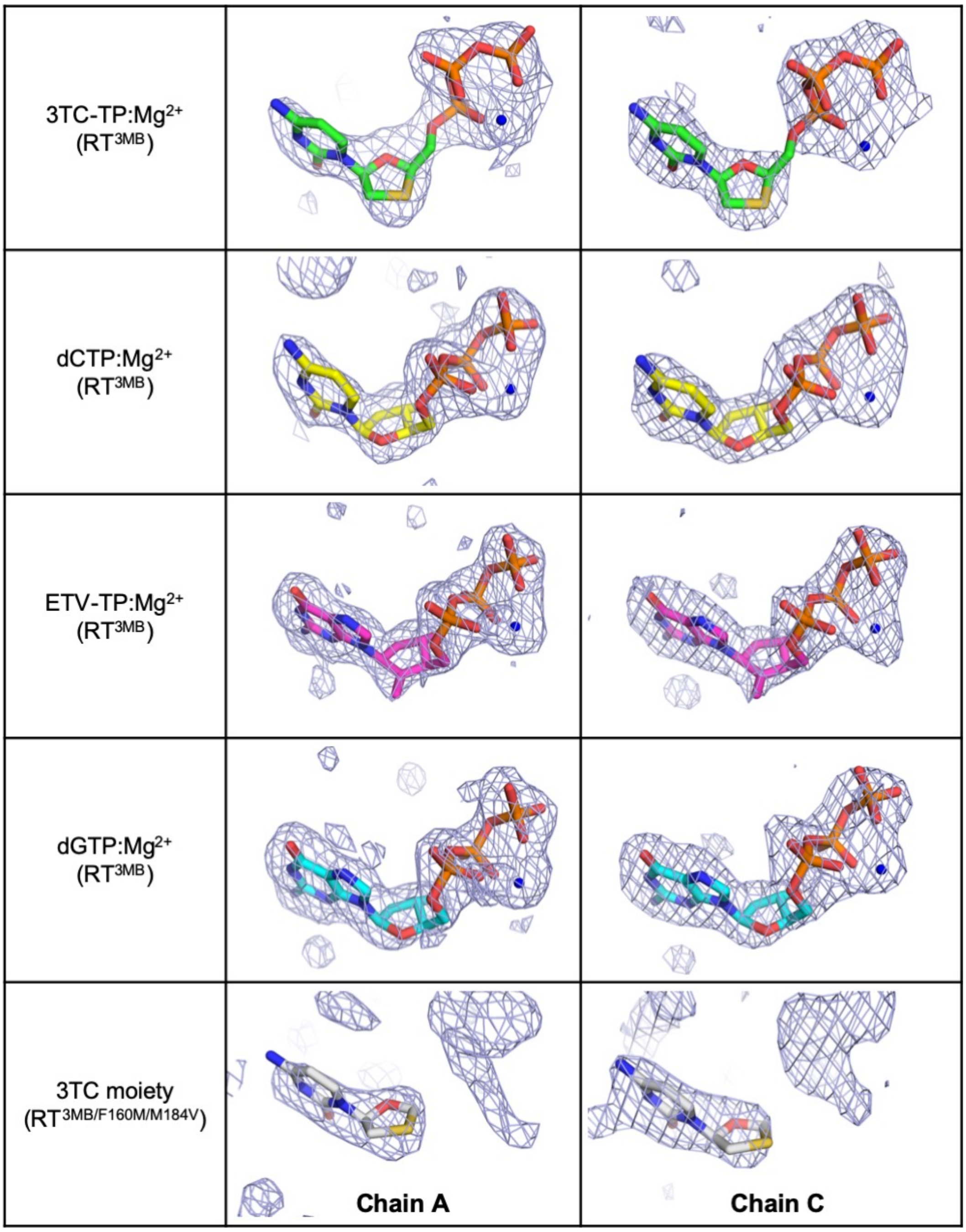

Supplementary Fig. S4. Simulated annealing $F_o - F_c$ omit map for the bound NRTIs/dNTP and Mg^{2+} for all chains analyzed in this study. All maps are contoured at 2.5σ level.

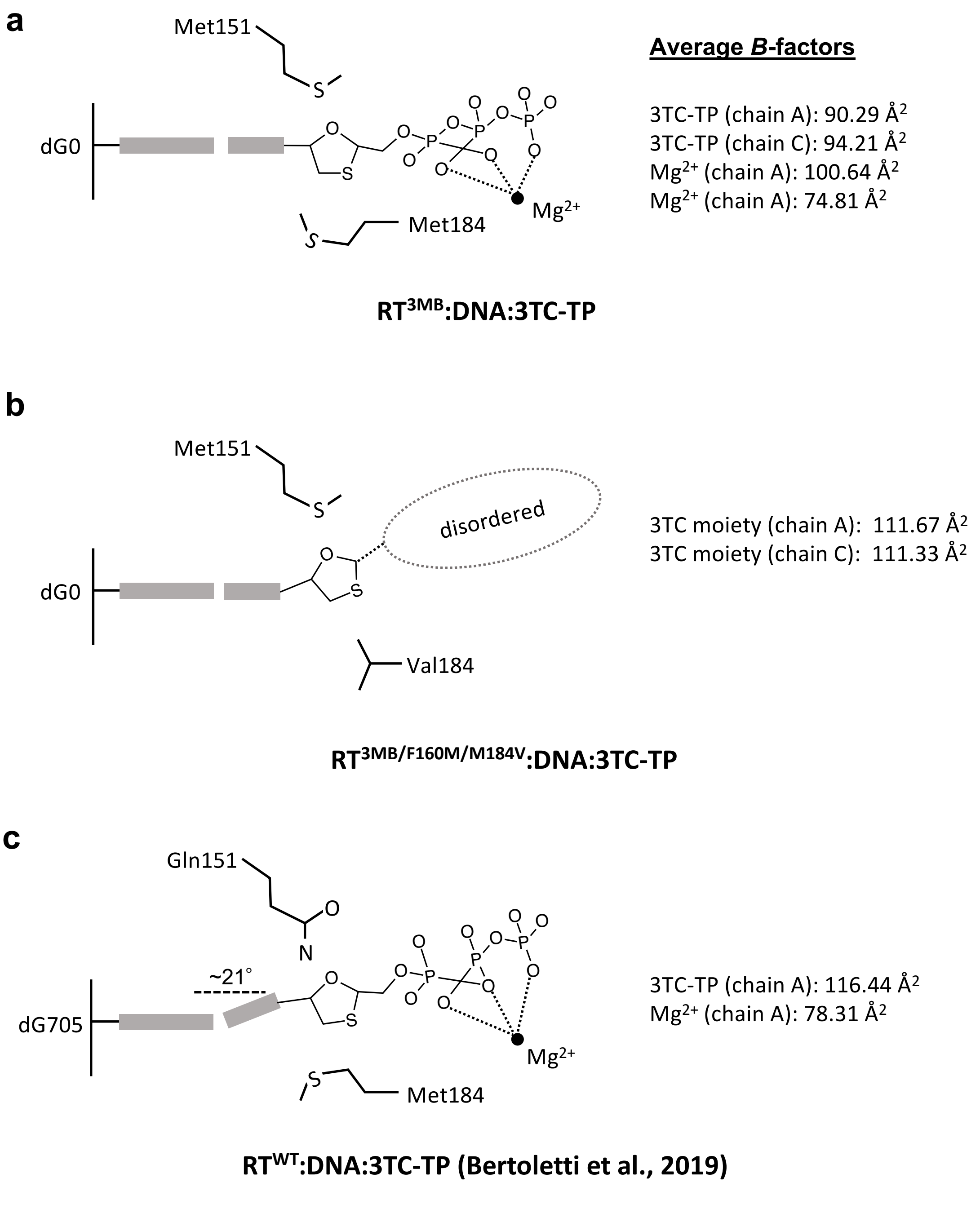

Supplementary Fig. S5. Schematic diagram showing the relative location of the bound 3TC-TP in the RT^{3MB} (a), $RT^{3MB/F160M/M184V}$ (b), and recently reported RT^{WT} by Bertoletti *et al.* (c)[34]. The base-pairings of dG, residues 151, 184 and the Mg^{2+} are also described. The average *B*-factors for 3TC-TP and Mg^{2+} in each structure are also indicated.

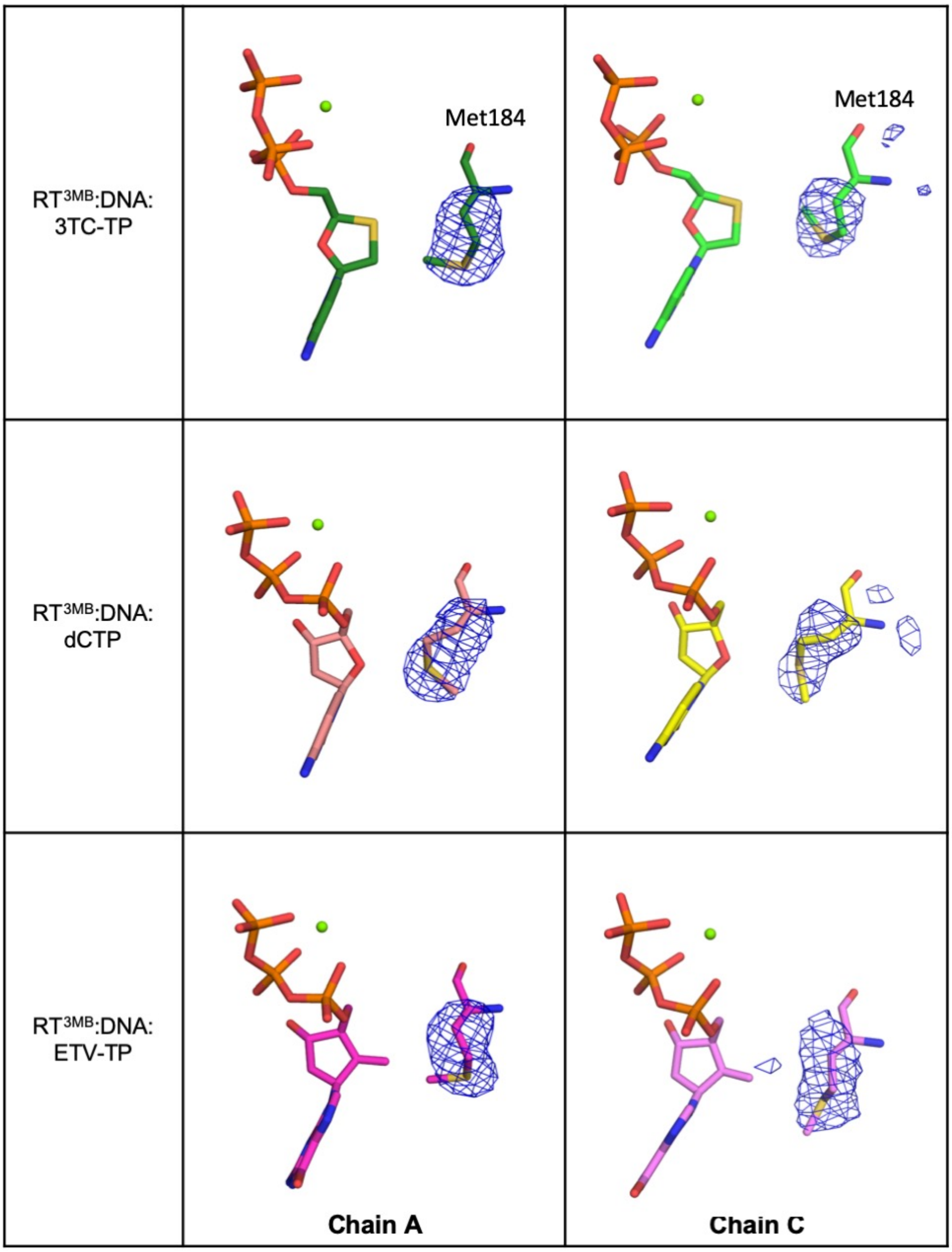

RT3MB:DNA:
3TC-TP
Met184
Met184
RT3MB:DNA:
dCTP
RT3MB:DNA:
ETV-TP
Chain A
Chain C

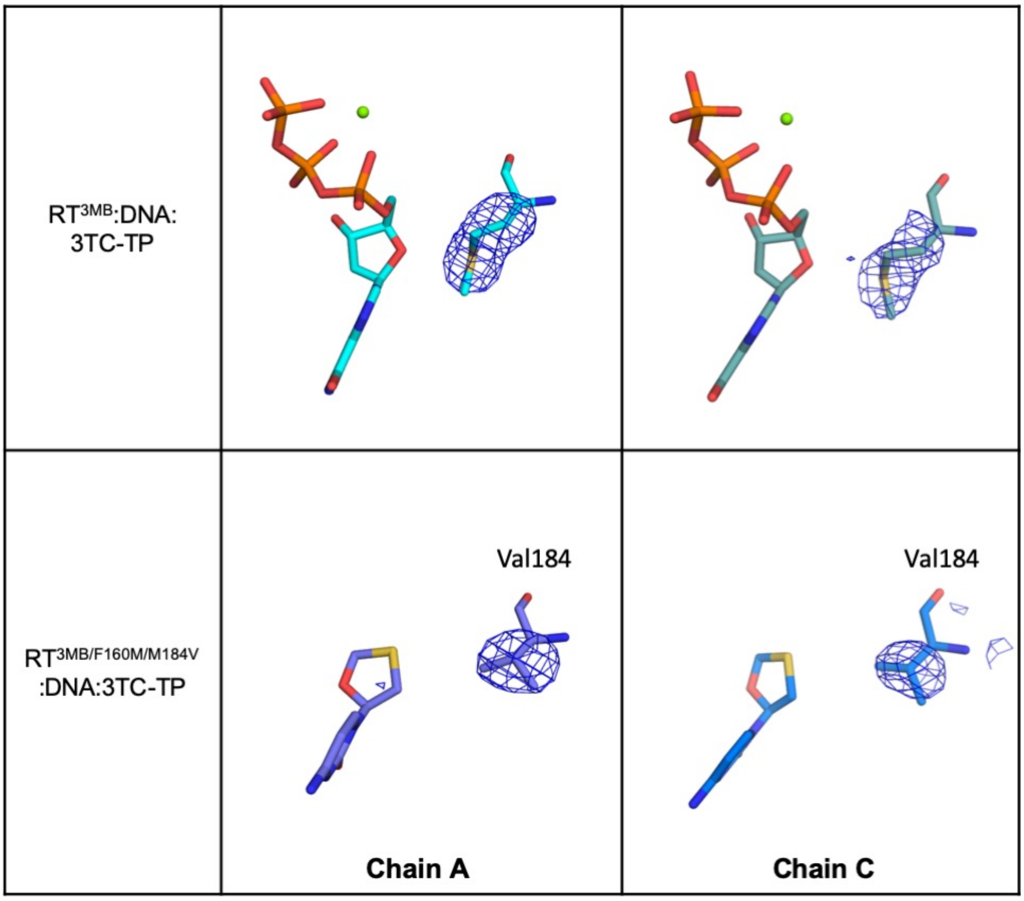

Supplementary Fig. S6. Simulated annealing $F_o - F_c$ omit map for the Met/Val184 side chains. The nearby NRTIs/dNTPs and Mg^{2+} are also shown. The maps are contoured at 3.0σ level.

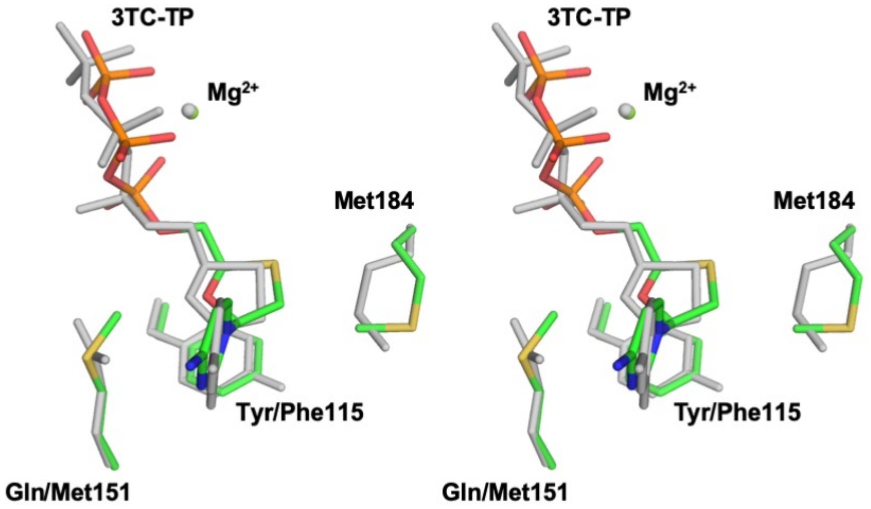

Supplementary Fig. S7. Stereo-view superimposition of the bound 3TC-TP, Mg^{2+} and the nearby residues (Gln/Met151, Tyr/Phe115 and Met184) of the present HIV-1 RT^{3MB}:DNA:3TC-TP and the recently reported HIV-1 RT^{WT}:DNA:3TC-TP[34]. The model for the RT^{WT}:DNA:3TC-TP is colored in gray.
